# Beating Naive Bayes at Taxonomic Classification of 16S rRNA Gene Sequences

**DOI:** 10.3389/fmicb.2021.644487

**Published:** 2021-06-18

**Authors:** Michal Ziemski, Treepop Wisanwanichthan, Nicholas A. Bokulich, Benjamin D. Kaehler

**Affiliations:** ^1^Laboratory of Food Systems Biotechnology, Institute of Food, Nutrition, and Health, ETH Zürich, Zurich, Switzerland; ^2^School of Science, University of New South Wales, Canberra, ACT, Australia

**Keywords:** microbiome, metagenomics, marker-gene sequencing, taxonomic classification, machine learning, neural networks

## Abstract

Naive Bayes classifiers (NBC) have dominated the field of taxonomic classification of amplicon sequences for over a decade. Apart from having runtime requirements that allow them to be trained and used on modest laptops, they have persistently provided class-topping classification accuracy. In this work we compare NBC with random forest classifiers, neural network classifiers, and a perfect classifier that can only fail when different species have identical sequences, and find that in some practical scenarios there is little scope for improving on NBC for taxonomic classification of 16S rRNA gene sequences. Further improvements in taxonomy classification are unlikely to come from novel algorithms alone, and will need to leverage other technological innovations, such as ecological frequency information.

## Introduction

Microbial communities are integral components of diverse ecosystems on planet Earth, supporting both environmental and human health (The Human Microbiome Project Consortium, 2012; Thompson et al., 2017). Investigating the role of microorganisms in these environments often involves characterizing the composition of these communities using high-throughput DNA sequencing methods, most commonly of universal marker genes, such as small subunit rRNA genes (Thompson et al., 2017). Even short sequences (e.g., as obtained from “second-generation” sequencing instruments) of 16S rRNA gene hypervariable domains can differentiate bacterial families and genera (Liu et al., 2008), making these marker genes popular targets for microbial census studies.

A critical step in any microbial census study is the taxonomic classification of observed DNA sequences, to infer the relative abundance of different taxonomic groups. This is performed by comparison of observed sequences to a reference database of sequences from known taxa, using an appropriate taxonomic classifier (Robeson et al., 2020). A large number of taxonomic classification methods have been developed and benchmarked for classification of marker gene sequences (Bokulich et al., 2018b; Gardner et al., 2019), but among the most successful and ubiquitous in microbiome studies have been naive Bayes classifiers (NBC). The primacy of NBC was established by the Ribosomal Database Project (RDP) classifier (Wang et al., 2007), which utilized an NBC and demonstrated that genus-level accuracy could be achieved from short 16S rRNA gene sequences. The superiority of NBC for marker-gene sequence classification has proven robust over time, as we have shown more recently with the various classifiers implemented in q2-feature-classifier (Bokulich et al., 2018b), a taxonomic classification plugin for the popular QIIME 2 microbiomics software platform (Bolyen et al., 2019). Furthermore, we demonstrated that the accuracy of NBC could be significantly enhanced by providing ecological information about the expected frequency of different taxonomic groups in specific natural environments (Kaehler et al., 2019) to enable more reliable species-level classification of 16S rRNA gene sequences. This improves upon the assumptions of earlier NBC for marker-gene sequences (e.g., RDP classifier), which assume uniform class weights, i.e., that microbial species are equally likely to be observed.

Newer methods for taxonomic classification have been developed and tested, but have failed to reliably exceed the accuracy of NBC for marker-gene taxonomic classification, both in individual benchmarks (Lu and Salzberg, 2020) and in independent benchmarks (Almeida et al., 2018; Gardner et al., 2019). Notably, all benchmarks to date (except those in Kaehler et al., 2019) have tested NBC with uniform class weights, underlining that naive Bayes remains most accurate even without full optimization for specific sample types. This led us to consider three questions in the current study:

1. Could taxonomic frequency information benefit other taxonomic classifiers?
2. Could newer supervised learning algorithms exceed the accuracy of NBC?
3. Do decreasing performance advances in the microbiome taxonomy classification literature indicate that we are reaching an upper limit of performance for classification of short marker-gene sequences?

Class weight information can be utilized by a variety of supervised classification methods, so we hypothesized that using class weights could provide these methods with a much-needed performance boost. We chose two newer machine learning classification algorithms that have been successfully applied to other problems in bioinformatics (e.g., sample classification, e.g., Bokulich et al., 2016, 2018a; Roguet et al., 2018), but little-explored for DNA sequence annotation: Random Forests (RF) (Breiman, 2001) and convolutional neural networks (CNN) (Lecun et al., 1998). These algorithms have shown favorable performance against the RDP classifier in isolated tests (Chaudhary et al., 2015; Fiannaca et al., 2018; Busia et al., 2019; Zhao et al., 2020) but have not been independently benchmarked, nor compared against NBC with ecologically informed class weights.

We demonstrate that RF and CNN come close to but fail to exceed the accuracy of NBC when utilizing class weight information. Additionally, we use a “perfect” classifier to establish an upper bound for classification accuracy. We discover that, at least for short reads of 150 nt, there can be almost no improvement over an NBC if class weights are used. If longer reads are used (all of the V4 region) then there is limited scope for improvement, but again only if class weights are used. Finally, NBCs remain easier and faster to train than RF and CNN classifiers with fewer hardware requirements.

## Results

We selected RF and CNN classifiers as promising methods for DNA sequence taxonomy classification, due to promising performance of various implementations in recent isolated reports (Chaudhary et al., 2015; Fiannaca et al., 2018; Busia et al., 2019; Zhao et al., 2020). In particular, the use of ensemble classification by RF is a potentially attractive means of efficiently calculating class probabilities via random selection of sequence data in each decision tree. The ability of CNNs to learn complex patterns, and in particular to model spatial organization in sequence data (Busia et al., 2019), make CNNs promising for DNA sequence annotation tasks. We utilized a kmer bagging approach for feature extraction prior to both RF and NBC classification, as has been commonly implemented in NBC including the RDP classifier (Wang et al., 2007; Bokulich et al., 2018b). However, kmer bagging fails to leverage the mid- to long-range spatial organization of DNA sequences. Hence, we used a Word2Vec (Mikolov et al., 2013) encoding for feature extraction prior to CNN classification, similar to the spatial encoding schemes implemented for other CNN classifiers (Busia et al., 2019; Zhao et al., 2020).

### Random Forest Classifiers

We performed hyperparameter tuning of the RF classifiers following a two-tiered approach. Cross validation was performed on sequences in the Greengenes reference data set (McDonald et al., 2012) and on sample compositions derived from real samples downloaded from the Qiita database (Gonzalez et al., 2018). All of the tests of the NBC and RF classifiers that we performed used taxonomic weighting information (Kaehler et al., 2019).

First, a grid search was performed on a comparatively smaller data set to select hyperparameters with primary performance effects (all samples of 150 nt length labeled as sediment (non-saline) in Qiita on 20 March 2019 (Thompson et al., 2017), 188 samples, downloaded using q2-clawback (Kaehler et al., 2019) see Supplementary Material and Supplementary Table 1 for details). A second grid search was performed on a much larger animal distal gut data set (downloaded from Qiita on 23 May, 2019 with the same parameters, 22,454 samples). The results of the initial tests were that max_depth and max_features were the only classifier parameters that had a meaningful impact on classification accuracy. Except for confidence, all parameters are those of the scikit-learn classifier (see section “Materials and Methods”). Additionally, a confidence parameter of 0.7 was found to give greater accuracy than a confidence parameter of 0.9.

In all cases, classification accuracy was measured using F-measure for species-level classification. We also tested final results using the Matthews correlation coefficient (MCC) (Chicco and Jurman, 2020). MCC was chosen to reduce the reported bias in F-measure in the presence of imbalanced classes. In all cases results were qualitatively the same. For the same confidence level, parameter choice also gave the same rankings for MCC as F-measure (see Supplementary Figure 1).

**FIGURE 1:**
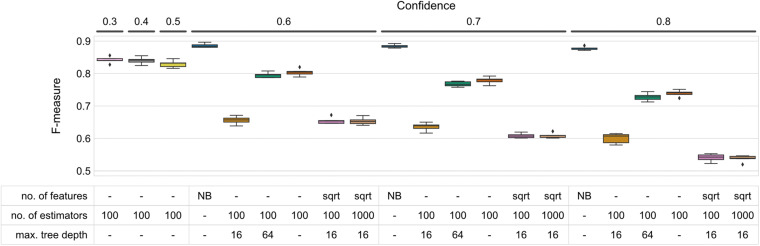
F-measure accuracy performance of RF and NB classifiers. Box-and-whisker plots indicate the median and quartile distributions of F-measures for each classifier and configuration, across 5-fold of CV. RF classifier configurations were tested via a grid search across the hyperparameters listed in the subset table. NB classifiers do not have equivalent parameters, and hence only NB is listed in the table beneath bars representing NB classifiers. Both RF and NB classifiers were tested at multiple confidence levels. None of the tested parameter sets outperformed the NBC at any of the confidence levels (Wilcoxon signed-rank test *p* < 0.05).

The parameters selected for the second tier of parameter tuning are shown in Table 1. A full grid search testing all combinations of parameters was not performed because of operational difficulties balancing requests for walltime, number of CPUs, and memory usage on a shared computational resource (see Supplementary Table 2 and Supplementary Figure 4) and negligible impacts on performance (Figure 1). If it is not possible to train a classifier on a machine with 14 CPUs and 3TB of memory in under 24 h, it is not useful to the wider community regardless of accuracy (Bokulich et al., 2020), and hence these configurations were not pursued further. A max_depth of None implies that nodes were expanded until all nodes were pure. max_features of None implies that the maximum number of features was the number of features.

**TABLE 1:** Parameter values used for computationally intensive grid search on animal-distal-gut samples.

Parameter	Values		
n_estimators	100	1,000	–
max_depth	16	64	None
max_features	sqrt	None	–
Confidence	0.6	0.7	0.8

Results indicate that maximum tree depth (max_depth) exerted the greatest influence on classification accuracy (Figure 1 and Supplementary Figure 1). Regardless of the confidence level, increasing the maximum depth leads to an increase in F-measure. The most significant change can be observed between 16 and 64 nodes (average F-measures of 0.636 ∓ 0.006 and 0.768 ∓ 0.004 at confidence 0.7, respectively, standard error measured over folds). Increasing max_depth beyond 64, however, does not lead to an appreciable increase in accuracy, and using unlimited tree depth (i.e., max_depth = None; tree nodes are expanded until leaf purity is achieved) yields marginally higher F-measures at all confidence levels (*F* = 0.779 ∓ 0.005 at confidence 0.7) (Figure 1).

Decreasing the number of features (max_features) to be taken into account while deciding on node splitting resulted in a modest decrease in classification accuracy [0.636 ∓ 0.006 and 0.608 ∓ 0.003 average F-measure for using all of the features as compared to sqrt (number of features)]. This performance decrease was least pronounced at lower confidence levels. Similarly, increasing the number of estimators (n_estimator, i.e., trees in the forest) had no or very low influence on classification accuracy, regardless of the confidence level. Increasing the number of estimators from 100 to 1,000 reliably caused memory issues, however, particularly with a maximum tree depth of 64 (see Supplementary Table 2).

None of the parameter sets tested in our study outperformed the NBC at any of the confidence levels we tested (Wilcoxon signed-rank *p* < 0.05). To test whether reducing the classification confidence threshold further beyond the level of 0.6 could help increase RF’s performance, we trained and evaluated an additional set of classifiers with fixed parameters (max. number of features, 100 estimators, max. tree depth) while varying confidence in the range 0.3–0.5. Decreasing the confidence marginally increased the test set’s recall and F-measures at the cost of precision (Figure 1 and Supplementary Figure 1), however the accuracy achieved by the NBC could still not be obtained (Figure 1).

Interestingly, precision of the RF classifier tested with most of the parameter sets could in many cases outperform the NB model and it was rather insensitive to parameter changes given a confidence level (Supplementary Figure 1, top panel). It was the recall, however, that not only varied greatly between parameter sets, but also could never come close to that of the NB (Supplementary Figure 1, bottom panel).

### Convolutional Neural Networks

Following our tests of RF classifiers, we were interested in evaluating whether we could leverage recent advances in neural network-based models for superior taxonomic classification. Cross validation was performed as described for RF. To reduce run time, we used a relatively small data set that consisted of all 5,632 of the animal distal gut 150 nt samples that were available from Qiita on 1 June, 2018 (downloaded using q2-clawback; Kaehler et al., 2019).

More specifically, we focused on CNNs as their performance is favorable in the literature (Chaudhary et al., 2015; Fiannaca et al., 2018; Busia et al., 2019; Zhao et al., 2020) and for their relatively parsimonious parameterization and insensitivity to insertion and deletion events. Before feeding the DNA sequences to the network, we applied the Word2Vec model in its Continuous-bag-of-words (CBOW) implementation to convert genetic information into a series of 300-element vectors. That not only allowed us to convert the k-mers into numerical values but also carried additional information about relatedness/similarity between any two k-mers within a given sequence.

**FIGURE 2:**
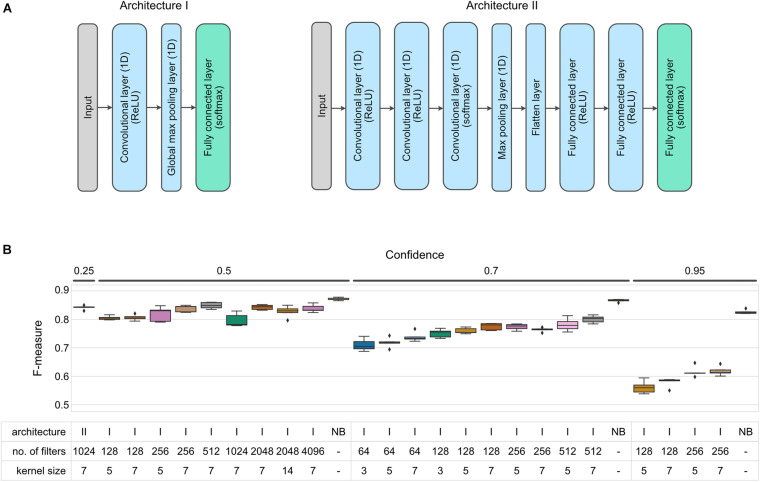
F-measure accuracy performance of CNN and NB classifiers. **(A)** CNN architectures implemented in this benchmark. **(B)** Box-and-whisker plots indicate the median and quartile distributions of F-measures for each classifier and configuration, across 5-fold of CV. CNN classifier configurations were tested via a grid search across the hyperparameters listed in the subset table. NB classifiers do not have equivalent parameters, and hence only NB is listed in the table beneath bars representing NB classifiers. Both CNN and NB classifiers were tested at multiple confidence levels. None of the tested networks outperformed the NBC at a given confidence level (Wilcoxon test *p* < 0.05).

For most of our tests we used a simple neural network with a single (one-dimensional) convolutional layer followed by a global max pooling layer and a classification layer (Figure 2A, architecture I). We varied the number of filters and kernel size of each filter (see Table 2) to test which of those parameters would have the greatest influence on the model performance (measured as Precision, Recall, F-measure, and MCC at the species level, similarly as was done for the Random Forest models).

**TABLE 2:** Parameter values used for grid search using the convolutional neural network.

Parameter	Values			
Filters	64	128	256	512
Kernel size	3	5	7	–
Confidence	0.5	0.7	0.95	–

Increasing either the number of filters or kernel size resulted in an increase of classification accuracy with average F-measures between 0.710 ∓ 0.009 and 0.801 ∓ 0.006 for models with filters = 64/kernel_size = 3 and filters = 512/kernel_size = 7, respectively (evaluated at 0.7 confidence level, Figure 2B and Supplementary Figure 2). Based on these initial findings, we selected a subset of parameter configurations to test the effect of confidence settings on CNN classification performance. Reducing the confidence parameter to 0.5 improved performance (average F-measure of 0.849 ∓ 0.005, filters = 512/kernel_size = 7), but further improvements were not observed when we further reduced confidence to 0.25 (see alternate model specifications in Supplementary Material).

We also attempted to improve performance by then extending the test range of number of filters and kernel size to 4,096 and 14 at a confidence level of 0.5 and found that doubling the number of filters or kernel size had little to no effect on the classifier accuracy (average F-measure of 0.843 ∓ 0.004, filters = 2,048/kernel_size = 7, 0.827 ∓ 0.009, filters = 2,048/kernel_size = 14). Finally, we tested a variety of different network architectures and two feature-extraction methods other than Word2Vec (see Supplementary Materials for details). One of the better results is represented by Architecture II in Figure 2B, which also used one-hot-encoding of individual nucleotides to build a sequence of vectors for input to the neural network.

While exhaustively testing all of the possible neural network architectures is not practical, a pattern emerged in our testing. That is that with tuning, it was possible to approach an average F-measure of around 0.85, but none of the models that we tested outperformed the NBC, which on the same data set with reads of the same length had an average F-measure of 0.866 ∓ 0.002 (all differences between CNN and NB results were statistically significant at *p* < 0.05 when evaluated at the same confidence level).

Comparing accuracy reported between F-measure and MCC, again the differences were qualitatively the same and different configurations were ranked almost identically within confidence levels.

Moreover, also in the case of CNN classifiers it is recall that plays a major role in differentiating between different parameter configurations (Supplementary Figure 2). While classification precision remained on an approximately similar level for most of the configurations tested, the recall increased as model complexity increased (in terms of model parameters). Regardless of the parameters used, however, CNN recall was always lower than that of NBC at a given confidence level (Supplementary Figure 2).

Finally, we were interested in checking whether the networks described above were prone to overfitting. Large model capacity (expressed as number of model parameters) with respect to the amount of training data can lead to the network learning features of the training set that are not universally relevant, thus reducing the accuracy when evaluating the model on the test set. We compared training histories of the architecture I with 512 filters and kernel size of 7 and architecture II with 1,024 filters and kernel size of 7 (Supplementary Figure 5). For all of the results that we report we trained for either 5 or 10 epochs (Supplementary Table 3), and overfitting was not evident at that stage in either of these examples.

### The Perfect Classifier

The underwhelming performance exhibited by RF and CNN classifiers led us to hypothesize that NBCs may already be approaching the upper limit of classification accuracy for this problem and hence alternative algorithms alone cannot exceed this performance. To test this hypothesis, we constructed a *perfect* classifier to measure the upper bound of classification accuracy for a given classification task. This classifier performs in-sample testing where the classifier can only fail if two or more species share exactly the same sequence. Where they do share the same sequence, one matching classification is chosen at random as the label for that sequence. The performance of such a classifier represents the upper limit of possible classification accuracy (Busia et al., 2019; Robeson et al., 2020).

We trained perfect classifiers with and without taxonomic class weighting to assess the upper bound of accuracy when using sequence information alone (uniform weights) or when leveraging ecological information. We also tested a range of confidences and for 150 nt amplicons or sequences that captured all of V4. See section “Materials and Methods” for implementation details of how confidence affected the perfect classifiers. We used the same data set that we had used for testing CNNs for this purpose.

It is an implementation detail of the CNN classifiers that variable length amplicons are difficult to handle, so our above tests truncated sequences at 150 nt. So comparing the 150 nt perfect classifiers first, average F-measures for perfect classifiers that used class weight information varied between 0.887 and 0.903. The best NBC with the same constraints achieved an average F-measure of 0.865 ∓ 0.002 (significantly different from the perfect classifier; Wilcoxon rank test *p* < 0.05). Note that the NBCs were performing out-of-sample cross validation whereas the perfect classifier is in-sample and therefore naturally inflated. While there is a small gap between these two figures, it certainly strongly limits scope for improvement over NBCs.

The story is slightly different if the perfect classifier was allowed to use all of the V4. In that case, where class weight information is utilized, the perfect classifier scored average F-measure between 0.934 and 0.943 whereas the similarly constrained NBC achieved 0.866 ∓ 0.002 (differences statistically significant at *p* < 0.05; Wilcoxon rank test). In other words, it performed roughly identically to where V4 was constrained to its first 150 nt. This is consistent with other empirical results (Liu et al., 2008; Bokulich et al., 2018b).

Interestingly, MCC gave slightly different results to F-measure for the perfect classifier for classifiers with high confidence levels (0.75 and 0.95). At these confidence levels, MCC was penalized with respect to the lower confidence levels. At lower confidence levels all differences were statistically significant when comparing the top-performing classifiers to “perfect” classifiers evaluated with similar parameters (Wilcoxon rank sum test, *p* < 0.05). As the purpose of the perfect classifier is to provide an upper bound on classification accuracy, however, the significant result indicates that additional optimization can only yield diminishingly small performance improvements.

**FIGURE 3:**
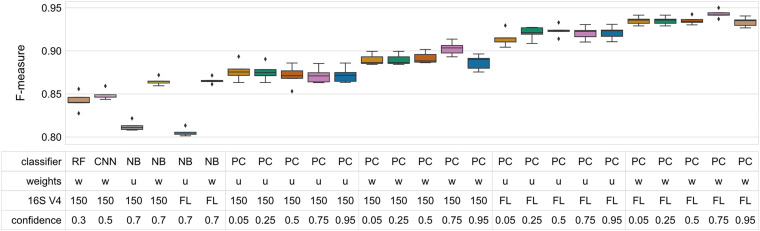
“Perfect” classifiers demonstrate the upper bound of classifier performance for V4 and full 16S rRNA gene sequences. These classifiers only fail when two species share an identical sequence, assuming uniform weights. Taxonomic weighting slightly increases classification accuracy both for V4 and full-length sequences. Box-and-whisker plots indicate the median and quartile distributions of F-measures for each classifier, across 5-fold of CV. The top-performing NB, RF, and CNN classifiers (trained and tested on V4 sequences) are compared to the “perfect” classifiers to demonstrate that the upper bound of performance is already being approached. All differences were statistically significant when comparing the top-performing classifiers to “perfect” classifiers evaluated with similar parameters (Wilcoxon rank sum test, *p* < 0.05). (Weights: w, weighted; u, uniform; 16S V4, 150–150 nt fragment of 16S rRNA V4 region; FL, all of the V4 region).

Finally, it is interesting to note the effect of incorporating class weight information on the NBC and perfect classifiers. For the NBC, the uniform classifiers (that did not use that information) performed almost the same for truncated and untruncated V4 sequences but were around 0.06 worse than when class weight information was used (average F-measure 0.812 ∓ 0.002 and 0.805 ∓ 0.002 respectively). For the perfect classifiers there was a clear progression where using all of V4 always improved accuracy and incorporating class weight information also increased accuracy. Class weight information did not improve classification accuracy for 150 nt sequences to match that of uniform classification on full V4 for the perfect classifier (Figure 3).

## Discussion

The goal of this study was to evaluate the utility of newer supervised learning techniques for taxonomic classification of 16S rRNA gene sequences, and in particular whether models based on convolutional neural networks could leverage ecological distribution information to match classification performance as shown previously for NBC (Kaehler et al., 2019). The implementations tested here managed to approach the classification accuracy of NBC, but even optimized CNNs could not match or exceed the performance of NBC, corroborating the recent findings of others (Zhao et al., 2020). Importantly, the goal of this study was not to test the exact implementations of RF or CNN classifiers developed by others (which have shown promising results but to our knowledge were not designed to leverage taxonomic weight information), but rather to evaluate the potential promise of advances in extracting taxonomic weight information (Kaehler et al., 2019) combined with spatial embedding of sequence information (Mikolov et al., 2013) for exceeding the taxonomic classification performance of NBC. Further independent benchmarks by others, and evaluation in more diverse test scenarios (e.g., non-16S rRNA gene targets) are warranted to further assess and optimize the performance of deep learning algorithms for taxonomic classification (Bokulich et al., 2020).

Following optimization of the hyperparameters evaluated in this study, RF was able to approach the accuracy performance delivered by NBC. Computational resources required to train this classifier, however, are substantially greater and could prohibit its practical application. Particularly large memory and computation time (<20 CPU hours vs. hundreds of CPU hours for NBC and RF, respectively) needed for training seemed to be a problem for some of the better-performing parameter sets due to a requirement to train many large trees (i.e., comprising many split nodes).

Given the best set of parameters and an optimized model architecture, CNN classifiers could approach, but not match, NBC accuracy performance. Moreover, training CNNs required a significant amount of computational resources and specific hardware, particularly in the case of more complex networks with many parameters. Our testing was only made feasible by employing modern graphics processing units (GPUs). GPUs are widely used to train neural networks, and this capacity has been suggested as an attractive feature of CNNs for taxonomic classification versus conventional methods (Busia et al., 2019). Even though training networks presented in this study required only a couple of hours (∼3 h) on a single GPU (NVIDIA GeForce RTX 2080) compared to ∼20 CPU hours for a typical NBC, GPUs can be considerably more expensive and difficult to configure and maintain, and hence are out of reach or less attractive to many researchers.

Our “perfect” classifier tests underline the fact that evolutionary conservation in most genetic targets for microbiome profiling limits the degree of taxonomic resolution that is possible, particularly when sequencing short marker-gene reads. Hence, mature, existing methods for classification (NBC and some alignment-based classifiers) have already neared the upper limits of classification accuracy. The relationship between read length, primer selection, marker-gene target, sequence entropy, and taxonomic resolution has been well documented for 16S rRNA genes and other common targets, and even with long sequence reads (e.g., full-length 16S rRNA genes) species-level resolution can be challenging for many clades (Wang et al., 2007; Liu et al., 2008; Bokulich et al., 2018b; Johnson et al., 2019; Robeson et al., 2020). This is in part complicated by muddled microbial taxonomies (Oren and Garrity, 2014; Yarza et al., 2014) and misannotations and other issues with reference databases used for taxonomic classification (Kozlov et al., 2016). Further improvements in taxonomy classification are unlikely to come from novel algorithms alone, and will require some combination of the following:

1. Use of spatial dependency in DNA sequences or other latent information. In spite of the current disappointing results, others have demonstrated the promise of spatially aware feature extraction prior to CNN classification for taxonomy or sample predictions (Busia et al., 2019; Zhao et al., 2020).
2. Use of ecological information from prior studies to hone classification accuracy (Kaehler et al., 2019).
3. Improvement of reference sequence and taxonomy databases (Parks et al., 2018; Robeson et al., 2020).
4. Longer read lengths and/or marker-gene targets (Johnson et al., 2019; Milani et al., 2020)
5. Improvements are not limited to accuracy, and could include more efficient classifiers with less runtime, memory, or other resource requirements (Bokulich et al., 2020).

We note that none of the methods compared in this work incorporated a feature selection step. It is possible that a feature selection step might increase performance of these methods (except for the perfect classifier), and warrants future investigation.

## Conclusion

Naive Bayes classifiers have demonstrated robust performance for taxonomic classification of DNA sequences for more than a decade (Wang et al., 2007), and recent improvements have further increased their accuracy (Bokulich et al., 2018b; Kaehler et al., 2019). Newer supervised learning methods such as neural networks offer exciting features with potential to further improve pattern recognition in microbiome data but so far have only demonstrated small or no improvements for taxonomic classification specifically (Zhao et al., 2020). In the current study, we find further evidence that NBCs remain supreme for taxonomic classification, even when applying taxonomic weighting and spatial encoding of sequence information, as well as hyperparameter tuning to optimize RF and CNN classifiers for 16S rRNA gene classification. It is worth noting that both RF and CNN classifiers comfortably outperform NBCs when they use taxonomic weighting information but the NBC does not. We demonstrate that NBCs are already nearing the performance limit of taxonomic classification of short 16S rRNA gene reads, indicating that further improvements will require technological and biological improvements or by leveraging other information (e.g., ecological observations) beyond sequence information alone. CNNs and other methods remain promising, however, and further optimization and benchmarking is warranted to fully assess the opportunities of deep learning techniques for microbial classification.

## Materials and Methods

### Random Forests

Cross validation of RF classifiers was performed using the methodology described in Kaehler et al. (2019). The RF classifier was tested using the standard q2-feature-classifier (Bokulich et al., 2018b) using a custom scikit-learn classifier specification to implement the scikit-learn random forest classifier (Pedregosa et al., 2011). Feature extraction was performed using the standard bag of overlapping 7-mers approach, also using scikit-learn. The code for the q2-feature classifier is available at https://github.com/qiime2/q2-feature-classifier. Cross validation code and classifier specifications are available at https://github.com/BenKaehler/paycheck.

Greengenes release 13_8 (McDonald et al., 2012) was used for the reference database and sample data was downloaded from Qiita (Gonzalez et al., 2018) using q2-clawback (Kaehler et al., 2019). 188 samples labeled as sediment (non-saline) were downloaded on 20 March 2019 and 22,454 samples labeled as animal distal gut were downloaded on 23 March 2019. These samples have been uploaded to Zenodo^1^.

### Convolutional Neural Networks

Cross validation of CNN classifiers was again performed using the methodology described in Kaehler et al. (2019). Neural networks were implemented using the Tensorflow library^2^ via the Keras interface^3^. Feature extraction was performed using the Word2Vec algorithm (Rehurek and Sojka, 2010). A fork of the standard q2-feature-classifier was necessary to accommodate Keras models and is available at https://github.com/BenKaehler/q2-feature-classifier. Cross validation code and classifier specifications are available at https://github.com/BenKaehler/paycheck.

Greengenes release 13.8 (McDonald et al., 2012) was used for the reference database and sample data was downloaded from Zenodo^4^. That data was the Qiita animal distal gut data originally used in Kaehler et al. (2019).

In the embedding step, sequences were trimmed to 150 nt. Each sequence was converted into a “sentence” of overlapping 7-mers, which were then used as input to the Word2Vec algorithm as implemented in Gensim (Rehurek and Sojka, 2010) to transform each sequence into a sequence of 144 length-300 vectors. A window of 5 words was used for training and the Common Bag of Words (CBOW) algorithm (Rehurek and Sojka, 2010) was selected. Those images were then presented to the various neural network models as described in “Results” section.

### Perfect Classifier

The perfect classifier used the same data set as the CNN experiments and tests were entirely in-sample. The perfect classifier tests were “cross validation” tests only in the sense that they used the same frozen, randomized test sets as the CNN experiments to reduce random variation between the two. Code for the perfect classifier is available at https://github.com/BenKaehler/paycheck.

“Perfect” classification was made possible by in-sample testing because every sequence that was used for testing had already been seen by the classifier. Sample weight cross validation was also in-sample, in that the same aggregate weights were used for every test, although we found that performing weight-wise out-of-sample testing did not make a qualitative difference to the results.

For each sequence, a list of taxa that matched that exact sequence was compiled. Weights for each taxon in the list were calculated using the taxonomic weighting information or by equally weighting taxa for uniform weights. In both cases the weights were normalized for each sequence. If one of the taxa’s weight was greater than the chosen confidence level, the taxon with the maximum weight was chosen. If two or more taxa had equal maximum weight (as most often happened in the uniform case), one was chosen at random. If the confidence level was not exceeded by any weights, weights were aggregated at the second lowest taxonomic level and the procedure was repeated until a potentially truncated taxon was assigned.

### Statistical Analysis

To assess whether the classification performance (expressed as F-measure) differs significantly between various models (i.e., random forest and convolutional neural network variations) and the Naive Bayes classifier, we employed a two-tailed Wilcoxon rank sum test (when comparing CNN to NB results where sample sets differed) and a two-tailed Wilcoxon signed rank test (for all other comparisons). The analysis was performed at α = 0.05 using all of the test samples available for a given model (combined across all the folds) followed by Hommel correction for multiple testing (Hommel, 1988).

Additionally, to account for the potential bias resulting from highly imbalanced classes we evaluated all the models using the Matthews correlation coefficient (MCC) (Chicco and Jurman, 2020). MCC metric was shown to be a more reliable metric as it assesses the entire confusion matrix (i.e., true positive and negative, false positives and negatives), proportionally to class size.

## Data Availability Statement

The original contributions presented in the study are included in the article/Supplementary Material, further inquiries can be directed to the corresponding author/s.

## Author Contributions

BK and NB conceived and designed experiments. TW and BK performed data retrieval and initial experiments. MZ, TW, BK, and NB performed the analysis and interpretation and wrote the manuscript. All authors reviewed and approved the final manuscript.

## Conflict of Interest

The authors declare that the research was conducted in the absence of any commercial or financial relationships that could be construed as a potential conflict of interest.

## Footnotes

1 https://zenodo.org/record/4361424#.X90hI1MzaV4

2 https://www.tensorflow.org/

3 https://keras.io/

4 https://zenodo.org/record/2548899#.X9rPG1MzaV4
