# Supplementary material for: Beating Naive Bayes at Taxonomic Classification of 16S rRNA Gene Sequences
Source: Front Microbiol. 2021 Jun 18;12:644487. doi: 10.3389/fmicb.2021.644487 (PMC8249850; doi:10.3389/fmicb.2021.644487)
Supplement: Supplementary file 1 [file Data_Sheet_1.pdf]

Supplementary Materials

Random Forest Sediment-Non-Saline Tests

An initial grid search was performed using a smaller set of 188 sediment-non-saline samples for taxonomic weight cross validation. The parameters tested are shown in Supplemental Table 1.

n_estimators did not have an obvious impact on the accuracy i.e., n_estimators = 100 performed slightly better than n_estimators = 10 both at 0.7 and 0.9 confidence with the average F-measure scores of 0.058 and 0.057 respectively. Amongst these parameters, max_depth exhibited the greatest effect on F-measure at both 0.7 and 0.9 confidence. At max_depth = 4, 8, and 16, the average F-measure scores were 0.00247, 0.1135 and 0.4487 respectively. min_sample_leaf had little effect on the performance of the classifiers both at 0.7 and 0.9 confidence with average F-measure values of 0.059 and 0.0572 respectively. The last parameter we tested was max_features. max_features also substantially contributed to the classifiers' performance. The results also showed that ‘sqrt’ is better than ‘log2’ at both confidence levels.

Tested Feature Extraction Methods for Neural Networks

In addition to Word2Vec, we tested two other methods of extracting features from DNA sequences for input to neural network classifiers. We tried one-hot-encoding individual nucleotides (or averaging the appropriate one-hot-encoded nucleotides for ambiguous characters). We also tried naively hashing 7-mers. Both methods resulted in a sequence of vectors for each genetic sequence. As for Word2Vec, neither method led to a method that outperformed NBCs.

Tested Neural Network Architectures

Model2.json
Model: "sequential_17"

Layer (type)	Output Shape	Param #
conv1d_14 (Conv1D)	(None, 138, 128)	268928
global_max_pooling1d_13 (Glo	(None, 128)	0
dense_13 (Dense)	(None, 5403)	696987

Total params: 965,915
Trainable params: 965,915
Non-trainable params: 0

```
_________________________________________________________________
None
Ben4.json
Model: "sequential_3"
_________________________________________________________________
Layer (type)                 Output Shape              Param #
=================================================================
global_max_pooling1d_3 (Glob (None, 4096)              0
_________________________________________________________________
dense_7 (Dense)              (None, 4096)              16781312
_________________________________________________________________
dense_8 (Dense)              (None, 4096)              16781312
_________________________________________________________________
dense_9 (Dense)              (None, 5000)              20485000
=================================================================
Total params: 54,047,624
Trainable params: 54,047,624
Non-trainable params: 0
_________________________________________________________________
None
Model12.json
Model: "sequential_18"
_________________________________________________________________
Layer (type)                 Output Shape              Param #
=================================================================
conv1d_13 (Conv1D)           (None, 142, 512)          461312
_________________________________________________________________
global_max_pooling1d_11 (Glo (None, 512)               0
_________________________________________________________________
dense_11 (Dense)             (None, 5403)              2771739
=================================================================
Total params: 3,233,051
Trainable params: 3,233,051
Non-trainable params: 0
_________________________________________________________________
None
gg.json
Model: "sequential_1"
_________________________________________________________________
Layer (type)                 Output Shape              Param #
=================================================================
conv2d_1 (Conv2D)            (None, 144, 1, 64)        1856
_________________________________________________________________
conv2d_2 (Conv2D)            (None, 138, 1, 32)        14368
```

```
_________________________________________________________________
flatten_1 (Flatten)          (None, 4416)              0
_________________________________________________________________
dense_1 (Dense)              (None, 4877)              21541709
=================================================================
Total params: 21,557,933
Trainable params: 21,557,933
Non-trainable params: 0
_________________________________________________________________
None
Ben12-3.json
Model: "sequential_4"
_________________________________________________________________
Layer (type)                 Output Shape              Param #
=================================================================
max_pooling1d_4 (MaxPooling1 (None, 148, 4)            0
_________________________________________________________________
flatten_4 (Flatten)          (None, 592)               0
_________________________________________________________________
dense_10 (Dense)             (None, 1666)              987938
_________________________________________________________________
dense_11 (Dense)             (None, 3333)              5556111
_________________________________________________________________
dense_12 (Dense)             (None, 5000)              16670000
=================================================================
Total params: 23,214,049
Trainable params: 23,214,049
Non-trainable params: 0
_________________________________________________________________
None
Model4.json
Model: "sequential_19"
_________________________________________________________________
Layer (type)                 Output Shape              Param #
=================================================================
conv1d_15 (Conv1D)           (None, 138, 256)          537856
_________________________________________________________________
global_max_pooling1d_14 (Glo (None, 256)               0
_________________________________________________________________
dense_14 (Dense)             (None, 5403)              1388571
=================================================================
Total params: 1,926,427
Trainable params: 1,926,427
Non-trainable params: 0
```

```
_________________________________________________________________
None
Ben11.json
Model: "sequential_3"
_________________________________________________________________
Layer (type)                 Output Shape              Param #   
=================================================================
flatten_1 (Flatten)          (None, 600)               0         
_________________________________________________________________
dense_4 (Dense)              (None, 1666)              1001266   
_________________________________________________________________
dense_5 (Dense)              (None, 3333)              5556111   
_________________________________________________________________
dense_6 (Dense)              (None, 5000)              16670000  
=================================================================
Total params: 23,227,377
Trainable params: 23,227,377
Non-trainable params: 0
_________________________________________________________________
None
Ben12-5.json
Model: "sequential_5"
_________________________________________________________________
Layer (type)                 Output Shape              Param #   
=================================================================
max_pooling1d_5 (MaxPooling1 (None, 146, 4)            0         
_________________________________________________________________
flatten_5 (Flatten)          (None, 584)               0         
_________________________________________________________________
dense_13 (Dense)             (None, 1666)              974610    
_________________________________________________________________
dense_14 (Dense)             (None, 3333)              5556111   
_________________________________________________________________
dense_15 (Dense)             (None, 5000)              16670000  
=================================================================
Total params: 23,200,721
Trainable params: 23,200,721
Non-trainable params: 0
_________________________________________________________________
None
Ben12-2.json
Model: "sequential_3"
_________________________________________________________________
Layer (type)                 Output Shape              Param #   
```

```
=================================================================
max_pooling1d_3 (MaxPooling1 (None, 149, 4)          0
_________________________________________________________________
flatten_3 (Flatten)          (None, 596)             0
_________________________________________________________________
dense_7 (Dense)              (None, 1666)            994602
_________________________________________________________________
dense_8 (Dense)              (None, 3333)            5556111
_________________________________________________________________
dense_9 (Dense)              (None, 5000)            16670000
=================================================================
Total params: 23,220,713
Trainable params: 23,220,713
Non-trainable params: 0
_________________________________________________________________
None
Ben3.json
Model: "sequential_4"
_________________________________________________________________
Layer (type)                 Output Shape            Param #
=================================================================
global_max_pooling1d_5 (Glob (None, 8192)            0
_________________________________________________________________
dense_5 (Dense)              (None, 8192)            67117056
_________________________________________________________________
dense_6 (Dense)              (None, 8192)            67117056
_________________________________________________________________
dense_7 (Dense)              (None, 5000)            40965000
=================================================================
Total params: 175,199,112
Trainable params: 175,199,112
Non-trainable params: 0
_________________________________________________________________
None
Model13.json
Model: "sequential_14"
_________________________________________________________________
Layer (type)                 Output Shape            Param #
=================================================================
conv1d_12 (Conv1D)           (None, 137, 2400)       5762400
_________________________________________________________________
global_max_pooling1d_11 (Glo (None, 2400)            0
_________________________________________________________________
dense_18 (Dense)             (None, 2400)            5762400
```

```
_________________________________________________________________
dense_19 (Dense)             (None, 2400)              5762400
_________________________________________________________________
dense_20 (Dense)             (None, 5000)              12005000
=================================================================
Total params: 29,292,200
Trainable params: 29,292,200
Non-trainable params: 0
_________________________________________________________________
None
ModelX.json
Model: "sequential_20"
_________________________________________________________________
Layer (type)                 Output Shape              Param #
=================================================================
conv1d_4 (Conv1D)            (None, 138, 1024)         2151424
_________________________________________________________________
global_max_pooling1d_2 (Glob (None, 1024)              0
_________________________________________________________________
dense_2 (Dense)              (None, 5403)              5538075
=================================================================
Total params: 7,689,499
Trainable params: 7,689,499
Non-trainable params: 0
_________________________________________________________________
None

Ben1.json
Model: "sequential_5"
_________________________________________________________________
Layer (type)                 Output Shape              Param #
=================================================================
global_max_pooling1d_1 (Glob (None, 8192)              0
_________________________________________________________________
dense_2 (Dense)              (None, 5000)              40965000
=================================================================
Total params: 40,965,000
Trainable params: 40,965,000
Non-trainable params: 0
_________________________________________________________________
None
Model10.json
Model: "sequential_21"
_________________________________________________________________
```

```
Layer (type)                 Output Shape              Param #
=================================================================
conv1d_11 (Conv1D)           (None, 142, 128)          115328
_________________________________________________________________
global_max_pooling1d_9 (Glob (None, 128)               0
_________________________________________________________________
dense_9 (Dense)              (None, 5403)              696987
=================================================================
Total params: 812,315
Trainable params: 812,315
Non-trainable params: 0
_________________________________________________________________
None
Model1.json
Model: "sequential_22"
_________________________________________________________________
Layer (type)                 Output Shape              Param #
=================================================================
conv1d_13 (Conv1D)           (None, 140, 128)          192128
_________________________________________________________________
global_max_pooling1d_12 (Glo (None, 128)               0
_________________________________________________________________
dense_12 (Dense)             (None, 5403)              696987
=================================================================
Total params: 889,115
Trainable params: 889,115
Non-trainable params: 0
_________________________________________________________________
None
Ben8.json
Model: "sequential_2"
_________________________________________________________________
Layer (type)                 Output Shape              Param #
=================================================================
conv1d_4 (Conv1D)            (None, 144, 56)           1624
_________________________________________________________________
conv1d_5 (Conv1D)            (None, 144, 56)           3192
_________________________________________________________________
conv1d_6 (Conv1D)            (None, 144, 56)           3192
_________________________________________________________________
flatten_2 (Flatten)          (None, 8064)              0
_________________________________________________________________
dense_4 (Dense)              (None, 8064)              65036160
_________________________________________________________________
```

```
dense_5 (Dense)              (None, 8064)              65036160
_________________________________________________________________
dense_6 (Dense)              (None, 5000)              40325000
=================================================================
Total params: 170,405,328
Trainable params: 170,405,328
Non-trainable params: 0
_________________________________________________________________
None
s2v.json
Model: "sequential_3"
_________________________________________________________________
Layer (type)                 Output Shape              Param #
=================================================================
conv1d_3 (Conv1D)            (None, 140, 128)          192128
_________________________________________________________________
global_max_pooling1d_3 (Glob (None, 128)               0
_________________________________________________________________
dense_3 (Dense)              (None, 5403)              696987
=================================================================
Total params: 889,115
Trainable params: 889,115
Non-trainable params: 0
_________________________________________________________________
None
Model7.json
Model: "sequential_23"
_________________________________________________________________
Layer (type)                 Output Shape              Param #
=================================================================
conv1d_9 (Conv1D)            (None, 140, 64)           96064
_________________________________________________________________
global_max_pooling1d_7 (Glob (None, 64)                0
_________________________________________________________________
dense_7 (Dense)              (None, 5403)              351195
=================================================================
Total params: 447,259
Trainable params: 447,259
Non-trainable params: 0
_________________________________________________________________
None
Ben7.json
Model: "sequential_1"
_________________________________________________________________
```

```
Layer (type)                 Output Shape              Param #
=================================================================
conv1d_1 (Conv1D)            (None, 144, 28)           812
_________________________________________________________________
conv1d_2 (Conv1D)            (None, 144, 28)           812
_________________________________________________________________
conv1d_3 (Conv1D)            (None, 144, 28)           812
_________________________________________________________________
flatten_1 (Flatten)          (None, 4032)              0
_________________________________________________________________
dense_1 (Dense)              (None, 4032)              16261056
_________________________________________________________________
dense_2 (Dense)              (None, 4032)              16261056
_________________________________________________________________
dense_3 (Dense)              (None, 5000)              20165000
=================================================================
Total params: 52,689,548
Trainable params: 52,689,548
Non-trainable params: 0
_________________________________________________________________
None
Ben12-7.json
Model: "sequential_6"
_________________________________________________________________
Layer (type)                 Output Shape              Param #
=================================================================
max_pooling1d_6 (MaxPooling1 (None, 144, 4)            0
_________________________________________________________________
flatten_6 (Flatten)          (None, 576)               0
_________________________________________________________________
dense_16 (Dense)             (None, 1666)              961282
_________________________________________________________________
dense_17 (Dense)             (None, 3333)              5556111
_________________________________________________________________
dense_18 (Dense)             (None, 5000)              16670000
=================================================================
Total params: 23,187,393
Trainable params: 23,187,393
Non-trainable params: 0
_________________________________________________________________
None
ModelZ.json
Model: "sequential_24"
_________________________________________________________________
```

```
Layout (type)                Output Shape              Param #
```

```
Layer (type)                 Output Shape              Param #
=================================================================
conv1d_4 (Conv1D)            (None, 138, 4096)         8605696
_________________________________________________________________
global_max_pooling1d_2 (Glob (None, 4096)              0
_________________________________________________________________
dense_2 (Dense)              (None, 5403)              22136091
=================================================================
Total params: 30,741,787
Trainable params: 30,741,787
Non-trainable params: 0
_________________________________________________________________
None
Model6.json
Model: "sequential_25"
_________________________________________________________________
Layer (type)                 Output Shape              Param #
=================================================================
conv1d_4 (Conv1D)            (None, 138, 512)          1075712
_________________________________________________________________
global_max_pooling1d_2 (Glob (None, 512)               0
_________________________________________________________________
dense_2 (Dense)              (None, 5403)              2771739
=================================================================
Total params: 3,847,451
Trainable params: 3,847,451
Non-trainable params: 0
_________________________________________________________________
None

Ben9.json
Model: "sequential_11"
_________________________________________________________________
Layer (type)                 Output Shape              Param #
=================================================================
conv1d_31 (Conv1D)           (None, 144, 1024)         29696
_________________________________________________________________
conv1d_32 (Conv1D)           (None, 144, 1024)         1049600
_________________________________________________________________
conv1d_33 (Conv1D)           (None, 144, 1024)         1049600
_________________________________________________________________
max_pooling1d_7 (MaxPooling1 (None, 4, 1024)           0
_________________________________________________________________
flatten_7 (Flatten)          (None, 4096)              0
```

```
dense_25 (Dense)            (None, 4096)            16781312

dense_26 (Dense)            (None, 4096)            16781312

dense_27 (Dense)            (None, 5000)            20485000
=================================================================
Total params: 56,176,520
Trainable params: 56,176,520
Non-trainable params: 0

None
Model8.json
Model: "sequential_26"

Layer (type)                 Output Shape              Param #
=================================================================
conv1d_8 (Conv1D)            (None, 138, 64)           134464

global_max_pooling1d_6 (Glob (None, 64)                0

dense_6 (Dense)              (None, 5403)              351195
=================================================================
Total params: 485,659
Trainable params: 485,659
Non-trainable params: 0

None
Model3.json
Model: "sequential_27"

Layer (type)                 Output Shape              Param #
=================================================================
conv1d_16 (Conv1D)           (None, 140, 256)          384256

global_max_pooling1d_15 (Glo (None, 256)               0

dense_15 (Dense)             (None, 5403)              1388571
=================================================================
Total params: 1,772,827
Trainable params: 1,772,827
Non-trainable params: 0

None
```

```
Ben6.json
Model: "sequential_15"
_________________________________________________________________
Layer (type)                 Output Shape              Param #
=================================================================
global_max_pooling1d_12 (Glo (None, 8196)              0
_________________________________________________________________
dense_21 (Dense)             (None, 8196)              67182612
_________________________________________________________________
dense_22 (Dense)             (None, 8196)              67182612
_________________________________________________________________
dense_23 (Dense)             (None, 5000)              40985000
=================================================================
Total params: 175,350,224
Trainable params: 175,350,224
Non-trainable params: 0
_________________________________________________________________
None
ModelY.json
Model: "sequential_28"
_________________________________________________________________
Layer (type)                 Output Shape              Param #
=================================================================
conv1d_4 (Conv1D)            (None, 138, 2048)         4302848
_________________________________________________________________
global_max_pooling1d_2 (Glob (None, 2048)              0
_________________________________________________________________
dense_2 (Dense)              (None, 5403)              11070747
=================================================================
Total params: 15,373,595
Trainable params: 15,373,595
Non-trainable params: 0
_________________________________________________________________
None
Ben2.json
Model: "sequential_3"
_________________________________________________________________
Layer (type)                 Output Shape              Param #
=================================================================
global_max_pooling1d_4 (Glob (None, 8192)              0
_________________________________________________________________
dense_3 (Dense)              (None, 8192)              67117056
_________________________________________________________________
dense_4 (Dense)              (None, 5000)              40965000
```

```
=================================================================
Total params: 108,082,056
Trainable params: 108,082,056
Non-trainable params: 0
_________________________________________________________________
None
Model5.json
Model: "sequential_29"
_________________________________________________________________
Layer (type)                 Output Shape              Param #
=================================================================
conv1d_3 (Conv1D)            (None, 140, 512)          768512
_________________________________________________________________
global_max_pooling1d_1 (Glob (None, 512)               0
_________________________________________________________________
dense_1 (Dense)              (None, 5403)              2771739
=================================================================
Total params: 3,540,251
Trainable params: 3,540,251
Non-trainable params: 0
_________________________________________________________________
None
Ben5.json
Model: "sequential_6"
_________________________________________________________________
Layer (type)                 Output Shape              Param #
=================================================================
max_pooling1d_6 (MaxPooling1 (None, 4, 1024)           0
_________________________________________________________________
flatten_2 (Flatten)          (None, 4096)              0
_________________________________________________________________
dense_13 (Dense)             (None, 4096)              16781312
_________________________________________________________________
dense_14 (Dense)             (None, 4096)              16781312
_________________________________________________________________
dense_15 (Dense)             (None, 5000)              20485000
=================================================================
Total params: 54,047,624
Trainable params: 54,047,624
Non-trainable params: 0
_________________________________________________________________
None
Ben10.json
Model: "sequential_5"
```

```
_________________________________________________________________
Layer (type)                 Output Shape              Param #   
=================================================================
global_max_pooling1d_5 (Glob (None, 8192)              0         
_________________________________________________________________
dense_12 (Dense)             (None, 7000)              57351000  
_________________________________________________________________
dense_13 (Dense)             (None, 6000)              42006000  
_________________________________________________________________
dense_14 (Dense)             (None, 5000)              30005000  
=================================================================
Total params: 129,362,000
Trainable params: 129,362,000
Non-trainable params: 0
_________________________________________________________________
None
Model9.json
Model: "sequential_30"
_________________________________________________________________
Layer (type)                 Output Shape              Param #   
=================================================================
conv1d_10 (Conv1D)           (None, 142, 64)           57664     
_________________________________________________________________
global_max_pooling1d_8 (Glob (None, 64)                0         
_________________________________________________________________
dense_8 (Dense)              (None, 5403)              351195    
=================================================================
Total params: 408,859
Trainable params: 408,859
Non-trainable params: 0
_________________________________________________________________
None
Model6+.json
Model: "sequential_12"
_________________________________________________________________
Layer (type)                 Output Shape              Param #   
=================================================================
conv1d_2 (Conv1D)            (None, 138, 1024)         2151424   
_________________________________________________________________
global_max_pooling1d_2 (Glob (None, 1024)              0         
_________________________________________________________________
dense_4 (Dense)              (None, 1024)              1049600   
_________________________________________________________________
dense_5 (Dense)              (None, 1024)              1049600   
```

```
_________________________________________________________________
dense_6 (Dense)            (None, 5000)            5125000
=================================================================
Total params: 9,375,624
Trainable params: 9,375,624
Non-trainable params: 0
_________________________________________________________________
None
```

Supplemental Table 1. Parameter values used for initial grid search on sediment-non-saline samples.

Parameter	Values		
n_estimators	10	100	-
max_depth	4	8	16
min_sample_leaf	10	100	-
max_features	log2	sqrt	-
confidence	0.7	0.9	-

Supplemental Table 2. Resource usage for Random Forest classifier grid search. Failed rows exceeded requested resources and represent best attempts.

max_depth	n_estimators	max_features	confidence	CPUs	Memory (GB)	Walltime	Success
16	100	sqrt	0.6				
16	100	sqrt	0.7				
16	100	sqrt	0.8				
16	100	none	0.6				
16	100	none	0.7				
16	100	none	0.8	28	44.99	19:47:07	Complete
16	1000	sqrt	0.6	14	429.93	14:11:15	Complete

16	1000	sqrt	0.7	14	440.49	14:32:31	Complete
16	1000	sqrt	0.8	14	439.49	14:04:15	Complete
16	1000	none	0.6	14	234.88	41:34:36	Complete
16	1000	none	0.7	14	252.13	41:38:09	Complete
16	1000	none	0.8	14	246.25	41:44:51	Complete
64	100	sqrt	0.6	14	553.58	15:01:40	Complete
64	100	sqrt	0.7	14	543.31	15:40:53	Complete
64	100	sqrt	0.8	14	511.75	15:37:40	Complete
64	100	none	0.6	Missing	Missing	Missing	Complete
64	100	none	0.7	14	247.55	28:33:21	Complete
64	100	none	0.8	14	257.51	28:57:53	Complete
64	1000	sqrt	0.6	32	2775.04	5:47:25	Failed
64	1000	sqrt	0.7	32	2775.04	5:09:57	Failed
64	1000	sqrt	0.8	32	2764.8	5:10:47	Failed
64	1000	none	0.6	32	1341.44	24:00:27	Failed
64	1000	none	0.7	32	1341.44	24:00:51	Failed
64	1000	none	0.8	32	1341.44	24:00:34	Failed
none	100	none	0.6	14	381.99	42:33:51	Complete
none	100	none	0.7	14	391.19	42:37:42	Complete
none	100	none	0.8	14	406.15	42:23:27	Complete
none	1000	sqrt	0.6	32	2928.64	3:06:25	Failed
none	1000	sqrt	0.7	32	2938.88	3:10:45	Failed
none	1000	sqrt	0.8	32	2938.88	3:28:56	Failed
none	1000	none	0.6	32	1280	24:00:50	Failed
none	1000	none	0.7	32	1280	24:00:31	Failed
none	1000	none	0.8	32	1280	24:00:58	Failed

Supplemental Table 3. Model parameters for all tested convolutional neural networks.

Architecture	No. of filters	Kernel size	Confidence	No. of epochs
I	64	3	0.7	5
I	64	5	0.7	5
I	64	7	0.7	5
I	128	3	0.7	5
I	128	5	0.5, 0.7, 0.95	5
I	128	7	0.5, 0.7, 0.95	5
I	256	5	0.5, 0.7, 0.95	5
I	256	7	0.5	10
I	256	7	0.7, 0.95	5
I	512	5	0.7	5
I	512	7	0.5	10
I	512	7	0.7	5
I	1024	7	0.5	5
I	2048	7	0.5	5
I	2048	14	0.5	5
I	4096	7	0.5	5
II	1024	7	0.25	10

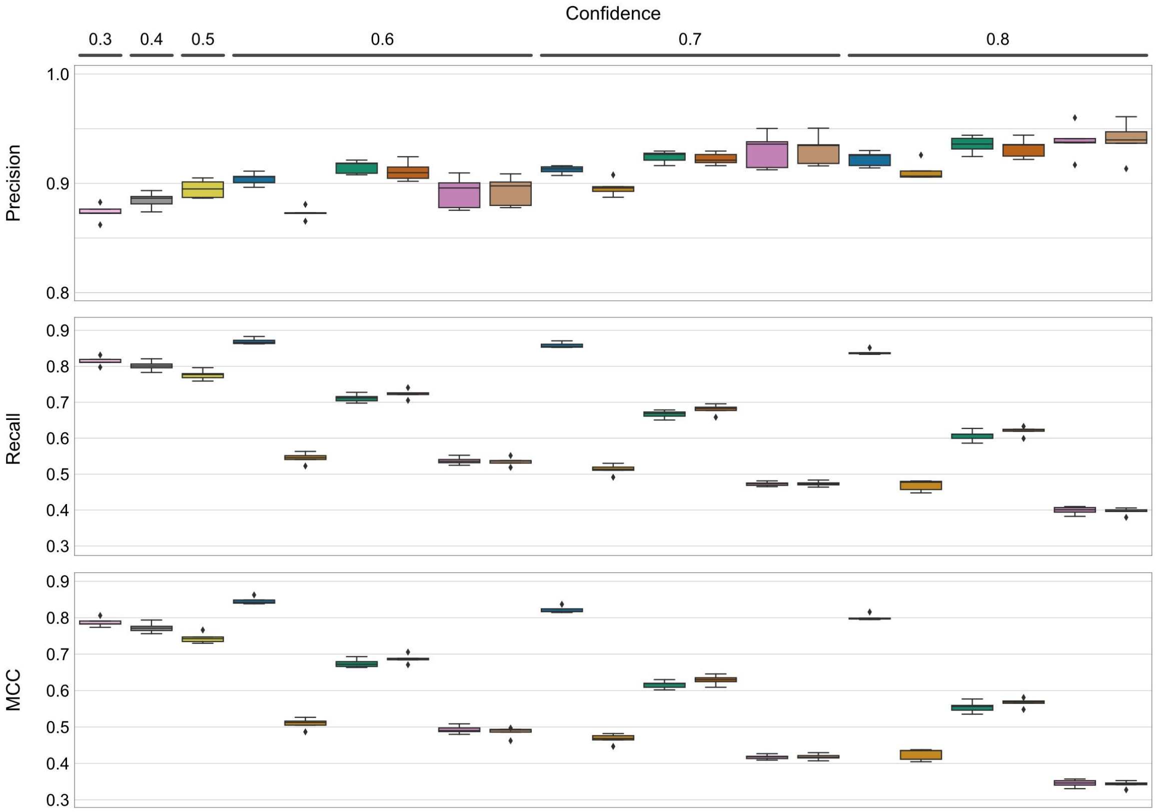

no. of features	-	-	-	NB	-	-	-	sqrt	sqrt	NB	-	-	-	sqrt	sqrt	NB	-	-	-	sqrt	sqrt
no. of estimators	100	100	100	-	100	100	100	100	1000	-	100	100	100	100	1000	-	100	100	100	100	1000
max. tree depth	-	-	-	-	16	64	-	16	16	-	16	64	-	16	16	-	16	64	-	16	16

Supplemental Figure 1. Precision, recall and Matthews correlation coefficient (MCC) of RF and NB classifiers. Box-and-whisker plots indicate the median and quartile distributions of precision, recall and MCC scores for each classifier and configuration, across 5 folds of CV. RF classifier configurations were tested via a grid search across the hyperparameters listed in the subset table. NB classifiers do not have equivalent parameters, and hence N/A is listed in the table beneath bars representing NB classifiers. Both RF and NB classifiers were tested at multiple confidence levels. None of the tested parameter sets outperformed the NBC at any of the confidence levels (Wilcoxon signed-rank performed on the MCC metric, p-value<0.05).

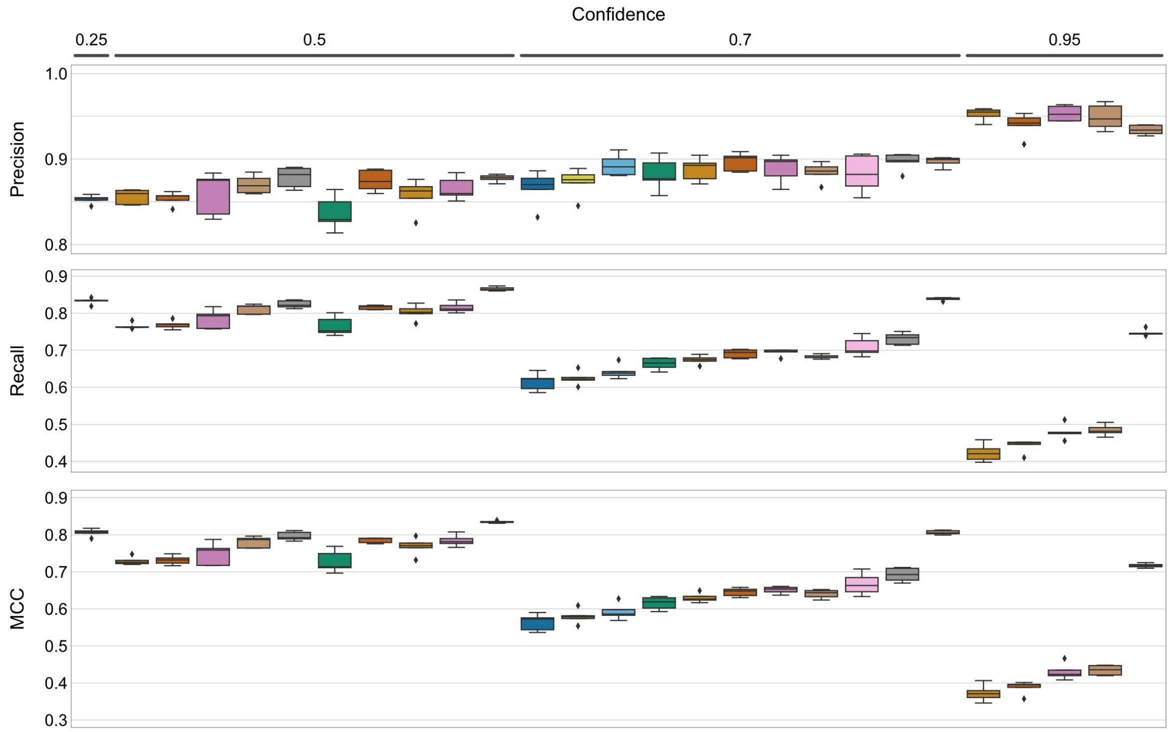

architecture	II	I	I	I	I	I	I	I	I	I	NB	I	I	I	I	I	I	I	I	I	I	NB	I	I	I	I	NB
no. of filters	1024	128	128	256	256	512	1024	2048	2048	4096	-	64	64	64	128	128	128	256	256	512	512	-	128	128	256	256	-
kernel size	7	5	7	5	7	7	7	7	14	7	-	3	5	7	3	5	7	5	7	5	7	-	5	7	5	7	-

Supplemental Figure 2. Precision, recall and Matthews correlation coefficient (MCC) of CNN and NB classifiers. Box-and-whisker plots indicate the median and quartile distributions of precision, recall, and MCC scores for each classifier and configuration, across 5 folds of CV. CNN classifier configurations were tested via a grid search across the hyperparameters listed in the subset table. NB classifiers do not have equivalent parameters, and hence N/A is listed in the table beneath bars representing NB classifiers. Both CNN and NB classifiers were tested at multiple confidence levels. None of the tested networks outperformed the NBC at a given confidence level (Wilcoxon test performed on the MCC metric, p-value<0.05).

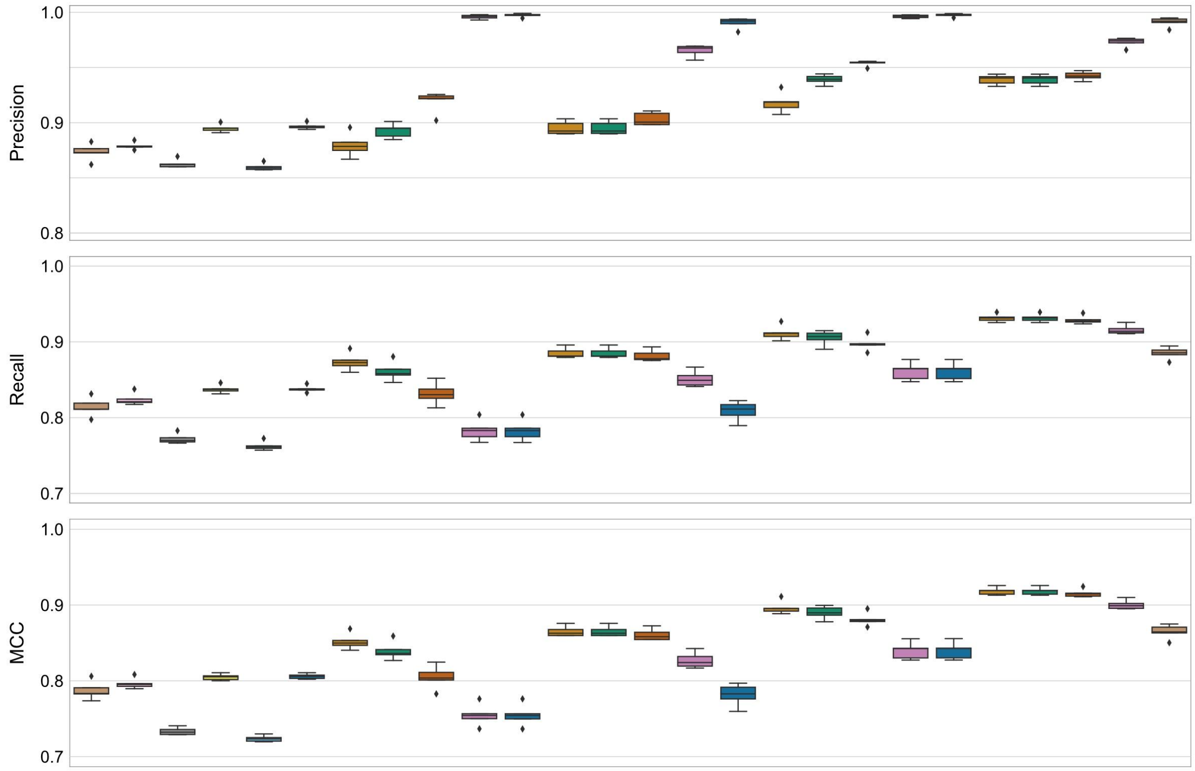

classifier	RF	CNN	NB	NB	NB	NB	PC	PC	PC	PC	PC	PC	PC	PC	PC	PC	PC	PC	PC	PC	PC	PC	PC	PC	PC	PC
weights	w	w	u	w	u	w	u	u	u	u	u	w	w	w	w	w	u	u	u	u	u	w	w	w	w	w
16S V4	150	150	150	150	FL	FL	150	150	150	150	150	150	150	150	150	150	FL	FL	FL	FL	FL	FL	FL	FL	FL	FL
confidence	0.3	0.5	0.7	0.7	0.7	0.7	0.05	0.25	0.5	0.75	0.95	0.05	0.25	0.5	0.75	0.95	0.05	0.25	0.5	0.75	0.95	0.05	0.25	0.5	0.75	0.95

Supplemental Figure 3. Precision, recall, and Matthews correlation coefficient (MCC) of Precision and recall of the top-performing RF, CNN, NB classifiers in comparison to the "perfect" classifier (PC). Box-and-whisker plots indicate the median and quartile distributions of precision, recall, and MCC scores for each classifier and configuration, across 5 folds of CV. All classifiers were tested at multiple confidence levels. All differences were statistically significant when comparing the top-performing classifiers to "perfect" classifiers evaluated with similar parameters (Wilcoxon rank sum test performed on the MCC metric, p-value<0.05), except of the uniform NB classifier trained on 150 nt sequences when compared to the uniform "perfect" classifier trained on the same sequences and evaluated at 0.75 confidence level. (Weights: w - weighted, u - uniform; 16S V4: 150 - 150 nt fragment of 16S rRNA V4 region, FL - all of the V4 region)

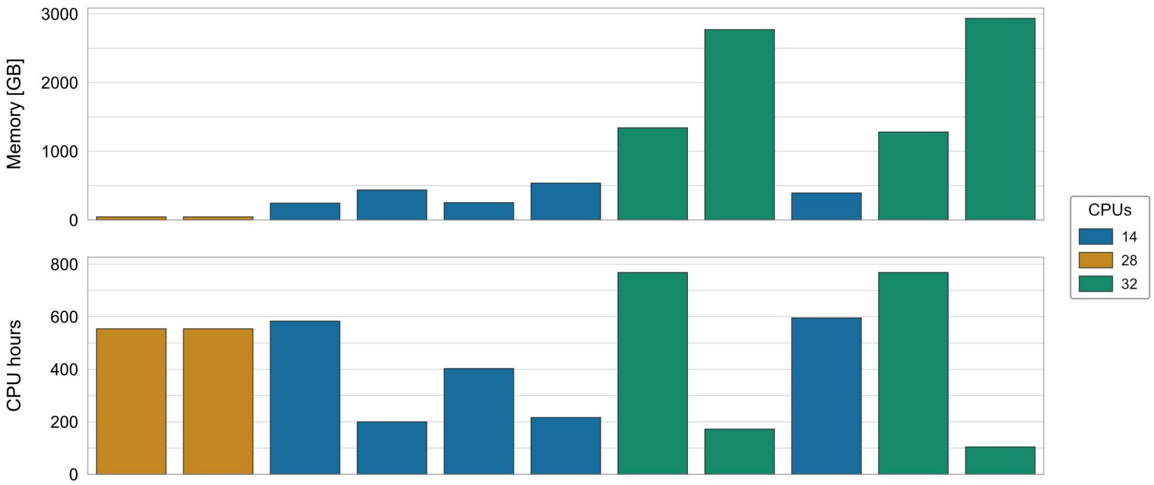

no. of features	-	sqrt	-	sqrt	-	sqrt	-	sqrt	-	-	sqrt
no. of estimators	100	100	1000	1000	100	100	1000	1000	100	1000	1000
max. tree depth	16	16	16	16	64	64	64	64	-	-	-
completion	yes	yes	yes	yes	yes	yes	no	no	yes	no	no

Supplemental Figure 4. Summary of computational resources required for training of the RF classifier using different parameter sets. As the forest complexity increases, the amounts of required volatile memory, CPUs and computation time increase too. The amount of time and CPU is expressed in “CPU hours” (where CPU hours = no. of CPUs × time in hours) that reflects the total amount of computation time, regardless of how many cores are being used. Configurations where completion is marked as “no” indicate runs that failed due to memory issues. Bars represent values averaged over 3 runs (at 3 confidence levels; compare Supplemental Table 2).

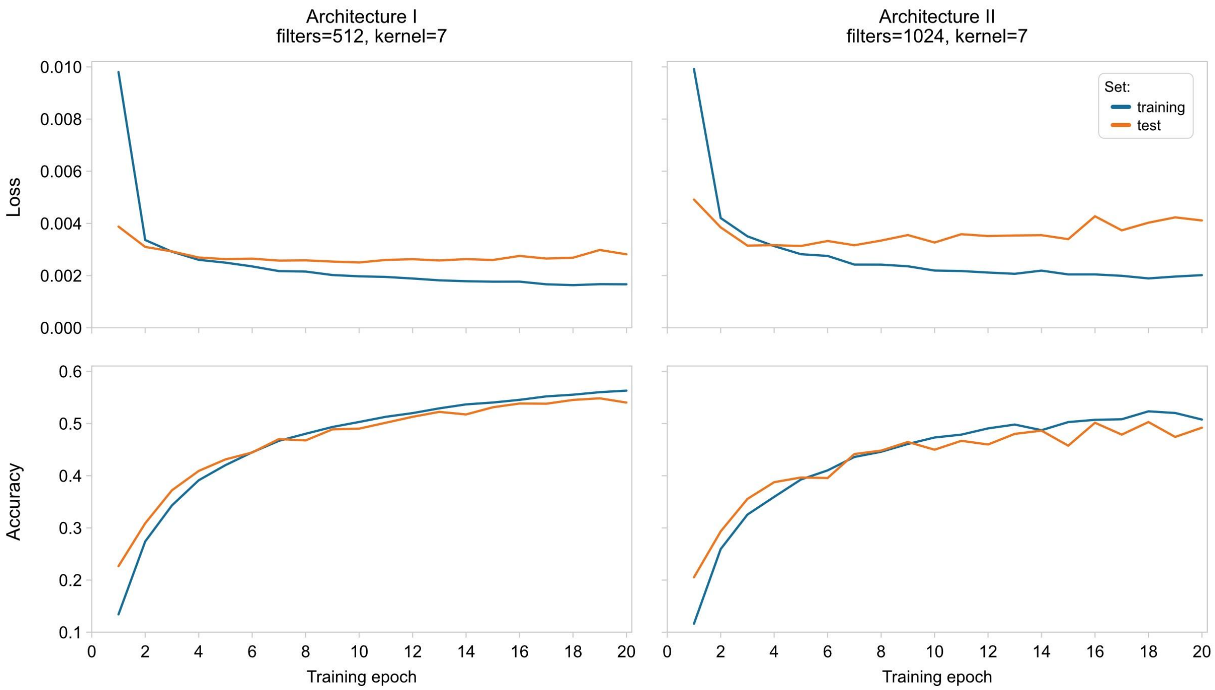

Supplemental Figure 5. Training histories of two CNN models with architecture I and II. The much more complex architecture II (the two plots on the right handside) shows signs of overfitting: test loss (top panels, orange curve) starts increasing around epoch 6 and continues rising until the end of training while the training loss (top panels, blue curve) remains unchanged. The simpler architecture I is less affected by overfitting.
